# Fabrication of uniform arrays of silver nanoparticles on silicon by electrodeposition in ethanol solution and their use in SERS detection of difenoconazole pesticide

**DOI:** 10.1039/d0ra08060h

**Published:** 2020-11-10

**Authors:** Tran Cao Dao, Truc Quynh Ngan Luong

**Affiliations:** Institute of Materials Science, Vietnam Academy of Science and Technology 18 Hoang Quoc Viet, Cau Giay 100000 Hanoi Vietnam nganltq@ims.vast.ac.vn; Graduate University of Science and Technology, Vietnam Academy of Science and Technology 18 Hoang Quoc Viet, Cau Giay 100000 Hanoi Vietnam

## Abstract

Surface-Enhanced Raman Scattering (SERS) is a technique currently widely used in the identification and quantification of organic and biological molecules at low concentrations, in which an important application is the detection of pesticide residues in food. To accomplish this task, SERS substrates with high Raman enhancement factor and good reproducibility are required. One of the most commonly used SERS substrates is the SERS substrate made of silver nanoparticles immobilized on a solid substrate. In this report we first present the results of electrochemical deposition of silver nanoparticles on the silicon surface using ethanol electrolyte solution. Thanks to both factors, electrochemical deposition (instead of electroless) and ethanol electrolyte (instead of aqueous), under optimal conditions, on the surface of silicon a monolayer of silver nanoparticles grew, which are uniform in shape and size and are arranged very close to each other with nanometer separation. Next we report on the use of fabricated arrays of silver nanoparticles in the role of a SERS substrate. To test the performance of the SERS substrate, the probe molecules used were molecules of difenoconazole, a well-known fungicide. Results showed that difenoconazole could be detected with a detection limit of 0.023 ppm (5.6 × 10^−8^ M).

## Abstract 2

An array of silver nanoparticles in a monolayer form with nanometer separation was electrodeposited onto silicon in an ethanol electrolyte and used as a SERS substrate in trace detection of difenoconazole pesticide.

## Graphical abstract

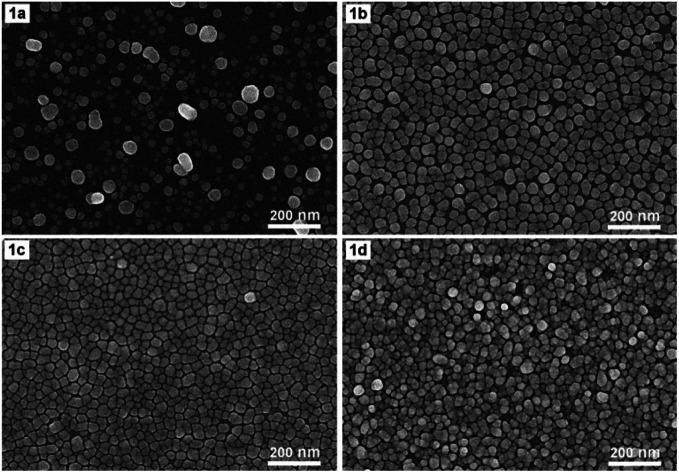

## Introduction

It is well known that each molecule has its own set of Raman scattering signals, which also means that each molecule can be identified based on these signals. This is also the main application of Raman spectroscopy. However, due to the weakness of the Raman scattering signal, the Raman spectrum of a molecule is only available when its concentration is high enough. Thank to surface-enhanced Raman scattering (SERS), which was discovered in 1977,^1,2^ this weakness has been overcome. With SERS, very small amounts of the analyte's molecules, in the concentration range of parts per million (ppm) to parts per billion (ppb), sometimes even smaller, can be detected. This result was achieved due to the presence of a nano-rugged metal surface. Such a surface acts as an amplifier of the Raman scattering signals of the analyte molecules that have been adsorbed onto it (or lie in very close proximity) and it is often referred to as a SERS substrate.

After an initial stage of actually using a nano-rugged metal surface as the SERS substrate, today an assembly of metal nanoparticles is the most typical type of the SERS substrate. These nanoparticles can be suspended in a colloidal solution, or immobilized on a certain solid substrate. However, if the nanoparticles are in suspension, to form a sample for Raman measurements they are also often mixed with the analyte, then covered on a flat substrate and allowed to dry. Therefore, the two types of SERS substrates made from the metal nanoparticles as mentioned above do not really differ much. In spite of this, it should be noted that when fixing nanoparticles on a solid substrate, we can arrange to create an ordered array of nanoparticles, thus creating a SERS substrate with better reproducibility. Regarding the metal materials for making SERS substrates, it is well known that silver and gold are the two best materials, of which silver is superior mainly because it has a better ability to allow a variety of different chemical and biological analytes to bind to its surface.^3^ For the above reasons, we chose to synthesize an array of silver nanoparticles (AgNPs) on a solid substrate, namely a flat silicon plate, for use as a SERS substrate.

Silicon has been chosen as the substrate for immobilizing onto it AgNPs for the following reasons. First of all, crystalline silicon in the form of wafers is an easy-to-find and relatively cheap material. Next, silicon is a neutral material for most analytes. Further, when used for SERS, silicon does not cause a luminescence background.^4^ It should also be emphasized that silicon is not merely a solid substrate to deposit AgNPs onto it like other materials. In the case when we use the method of reducing silver ions in a certain salt of silver to create AgNPs on silicon as in this study, then silicon plays the role of reducing agent. In practice, this often results in the fact that while AgNPs are deposited on silicon, the surface of the silicon itself is etched away, so that AgNPs firmly adhere to silicon.^5^

The first studies on fabrication of AgNPs on silicon for use as SERS substrates were published more than a dozen years ago.^6,7^ In these reports the authors synthesized AgNPs on silicon in a very simple way, by immersing the silicon wafers in an aqueous solution of silver nitrate (AgNO_3_) and hydrofluoric acid (HF). During this immersion plating process, AgNPs were deposited onto silicon through a galvanic replacement, where silicon was replaced by silver. More specifically, silver ions (in the aqueous solution of AgNO_3_) have been reduced to elemental silver by the action of silicon as a reducing agent, meanwhile silicon itself has been oxidized and dissolved in solution thanks to HF. Recently (2019) we also had a report of AgNPs on silicon that were fabricated in the same manner.^8^ Note that the coating of silicon surface by AgNPs with the above path can happen without using any electricity. However, we have found that if the coating is carried out under the action of a power source as an electrodeposition process, it will be able to better control the properties of the coating layer. In addition, we have observed that if the deposition is performed not in an aqueous electrolyte but in an ethanol-based electrolyte, then the obtained AgNPs will be more uniform in size and more ordered in the arrangement, especially they are very close to each other. Therefore in this work we will present the results of our study of the electrochemical deposition of an array of AgNPs on silicon (AgNPs@Si) in an ethanol-based electrolyte as well as the application of this AgNPs@Si array as a SERS substrate, with difenoconazole (DFC) pesticide used as the test compound.

We will now briefly describe why we have applied the synthesized AgNPs@Si SERS substrate for the DFC trace detection. As it is well known, one of the important uses of the SERS technique is the detection of pesticide residues in food.^9–11^ In modern agriculture, pesticides are widely used and in increasing amounts, especially in developing countries. This will result in an inevitable consequence that a small portion of the pesticide will remain in the food as residues. Pesticide residues will adversely affect the health of consumers and thus the need for its control arises. In addition to traditional methods such as different types of chromatography, it has been found that the SERS technique is also a good way to determine pesticide residues in food. In this study we will use the fabricated AgNPs@Si SERS substrates to detect trace concentrations of a specific pesticide, that is DFC. With the molecular formula of C_19_H_17_Cl_2_N_3_O_3_, DFC is a broad-spectrum triazole bactericide pesticide that acts by preventing the ergosterol biosynthesis and inhibiting steroid demethylation.^12–14^ This pesticide is widely used for crop protection against various fungi diseases such as gray mold, leaf spot, powdery mildew, and black spot.^13,14^ However, the abuse of DFC may cause negative effects on human health and the ecological environment. Several studies have reported that DFC may be associated with an increase in the incidence of hepatocellular adenomas and carcinomas for mice following long-term dietary exposure.^15,16^ The use of SERS technique to detect traces of DFC has been reported in the literature, but only in very few studies. In 2016 Huang and co-authors reported the use of colloidal gold nanoparticles purchased from OptoTrace Technologies to detect low-concentration DFC in pak choi extract.^13^ In that study the authors achieved a DFC detection limit of 0.413 mg L^−1^. In a more recent publication (2019) Wang *et al.* presented the results of the research on detecting low concentrations of DFC in standard solutions and grape extract using the SERS technique, with the SERS substrate made of silver coated gold nanoparticles aggregates.^14^ With this type of SERS substrate, the team has reached the limit of detection of DFC in the grape of 48 μg kg^−1^.

## Experimental

### Materials

The silicon used in this study was single-side polished, monocrystalline silicon with (100) orientation and 22 Ω cm resistivity, which was purchased from Semiconductor Wafer Inc. (Taiwan). Chemicals such as AgNO_3_ (99.8%) HF (40%), ethanol (99.7%), acetone (99.5%) and HNO_3_ (68%) were purchased from Xilong Chemical Co., Ltd. (Guangdong, China). The water used in the experiments was deionized water. The DFC used was in powder form with 99% purity, purchased from Sigma Aldrich.

### AgNPs@Si sample preparation and characterization

The preparation of AgNPs@Si samples was carried out as follows. First of all, the Si wafer was successively washed in ethanol (5 min), acetone (5 min), HNO_3_/H_2_O = 1/1 (10 min), 5% HF (5 min) and deionized water (several times) to remove organic contaminants. Next, a thin layer of aluminum was evaporated onto the backside of Si wafer (for later use as an ohmic contact during the electrodeposition of AgNPs on Si). Si wafer was then cut into 0.6 × 0.6 cm^2^ square samples, followed by electrochemical deposition of AgNPs on the polished surface of Si samples. The electrochemical system consists of two electrodes (connected *via* a DC power source) embedded in an electrolyte solution contained in a Teflon container. In this study the Si sample (with a thin layer of Al on the backside) was used as the negative electrode (or cathode), while the positive electrode (or anode) was a platinum grid. The two electrodes were placed in parallel with the distance between them being 2 cm. The electrolyte used was either aqueous or ethanolic solution of AgNO_3_ (0.1 mM) and HF (0.14 M). Silver electrodeposition on silicon was carried out for 4 minutes at a constant temperature of 17 °C in constant current mode, with a current density which is in the range of 0–0.5 mA cm^−2^.

Morphology and arrangement of the AgNPs formed on silicon were examined by scanning electron microscopy (SEM). The used electron microscope was S-4800 field emission scanning electron microscope (Hitachi, Japan). A few representative AgNPs@Si samples have been subjected to X-ray diffraction (XRD) analysis to check crystal structure and composition. XRD analysis was carried out on a XRD EQUINOX 5000 diffractometer (Thermo Scientific, France) using Cu Kα radiation.

### SERS measurements

As mentioned above, the DFC used in this study was DFC in powder form with 99% purity purchased from Sigma Aldrich. Raman spectrum of DFC powder and SERS spectra of low concentration DFC solutions adsorbed onto AgNPs@Si substrate were collected, using iRaman-Pro portable Raman spectrometer model BWS475-785H with 785 nm excitation laser (produced by B&W Tek, Inc., USA). This spectrometer provides a Raman spectrum over the range of 65 to 2800 cm^−1^ with a spectral resolution of better than 3.5 cm^−1^ and the excitation laser spot size is 105 μm (for the objective lens magnification of 20×). The preparation of samples for SERS measurement has been carried out as follows. First of all, the DFC powder was dissolved in ethanol to prepare a stock solution of 1000 ppm concentration. Next, this stock solution was diluted with deionized water to the concentrations to be analyzed, which are in the range of 0.1–100 ppm. Then, for each of the above concentrations, 25 μl of DFC solution was taken and dripped onto an AgNPs@Si substrate. After DFC solution dripping, samples were allowed to stand in air at room temperature until dry. SERS spectra of DFC with low concentrations were collected from these dried samples.

## Results and discussion

**Fig. 1:**
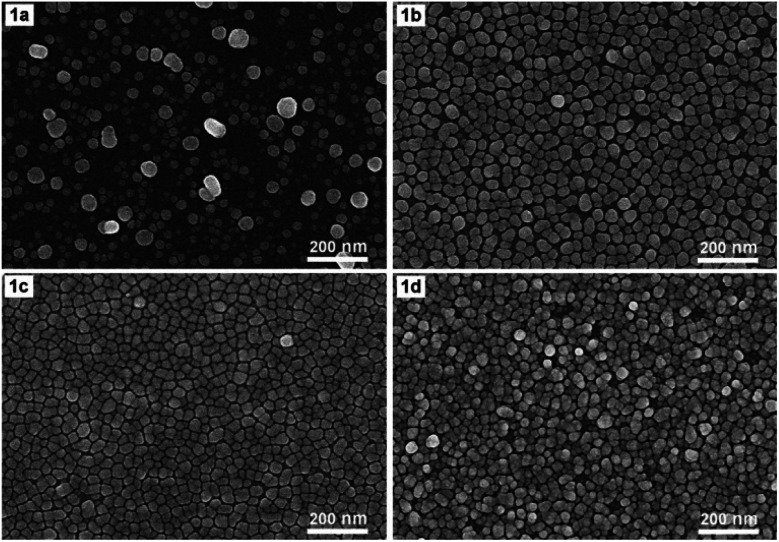
SEM images of AgNPs arrays that have been deposited onto Si samples at 17 °C for 4 minutes in the ethanol electrolyte solution containing 0.1 mM AgNO_3_ and 0.14 M HF with different current densities: 0 mA cm^−2^ (1a) (electroless deposition), 0.1 mA cm^−2^ (1b), 0.3 mA cm^−2^ (1c), and 0.5 mA cm^−2^ (1d).

Fig. 1 shows SEM images of AgNPs arrays that were formed on Si at 17 °C for 4 min in an ethanol solution containing 0.1 mM AgNO_3_ and 0.14 M HF either by electroless deposition (1a) or electrodeposition with a current density of 0.1 mA cm^−2^ (1b), 0.3 mA cm^−2^ (1c), and 0.5 mA cm^−2^ (1d), respectively. Fig. 1a shows that when the deposition is electroless, the resulting AgNPs are almost spherical or oblong in shape, but they are not uniform in size (which varies in the 10–60 nm region) together with not being on the same layer but belonging to different layers. Fig. 1b–d show the morphological changes of AgNPs when electrochemical deposition has been implemented in place of electroless. First of all, the shape and size of AgNPs become more uniform. Specifically, almost all particles are distorted spherical, with sizes varying in the range of 20–40 nm (Fig. 1b–d). Next, with the appropriate current density (0.1 or 0.3 mA cm^−2^), the AgNPs form an orderly array, with silver particles lying almost equally spaced on the same layer (Fig. 1b and c). It is worth noting that the distance between silver particles is very close, only within the nanometer region. Furthermore, this gap is smaller when the current density is 0.3 mA cm^−2^ (Fig. 1c) compared to that of the 0.1 mA cm^−2^ case (Fig. 1b). But when the current density increased to 0.5 mA cm^−2^, the silver particles were no longer on the same layer but overlapped in quite a mess (Fig. 1d).

As we mentioned in the Introduction section, when dipping a silicon wafer into a solution containing AgNO_3_ and HF, the silver ions will be reduced to the elemental silver by the action of silicon as a reducing agent. In turn, silicon is oxidized by HF to H_2_SiF_6_ – a soluble compound that will dissolve into the solution.^5^ There is also a second oxidation path, in which silicon is oxidized by H_2_O which is available in solution to SiO_2_, then this SiO_2_ will react with HF to form H_2_SiF_6_ which will dissolve into solution.^7^ The overall result is that while silver nanoparticles are created on the surface of the silicon wafer, the silicon itself on the surface is etched away. This is obviously a galvanic replacement process, in which silicon is replaced with silver. Galvanic replacement is one of the electroless deposition processes, it occurs spontaneously without the impact of external power. However, our results, as illustrated in Fig. 1, show that if we let the aforementioned redox process take place under the action of an appropriate constant current, then we can obtain an array of AgNPs with features that are more suitable for a SERS substrate. More specifically, it is an array of AgNPs with uniform shape and size that are arranged very close to one another (but without touching) on the same layer. Such a typical array is the one shown in Fig. 1c, which is obtained by electrodeposition of AgNPs on silicon with a current density of 0.3 mA cm^−2^ in the ethanol electrolyte. Note that due to the fact that the above redox process could take place even without an external power source, so when switching to electrodeposition only a very small current (0.3 mA cm^−2^) is required to create the AgNPs array with the best features.

**Fig. 2:**
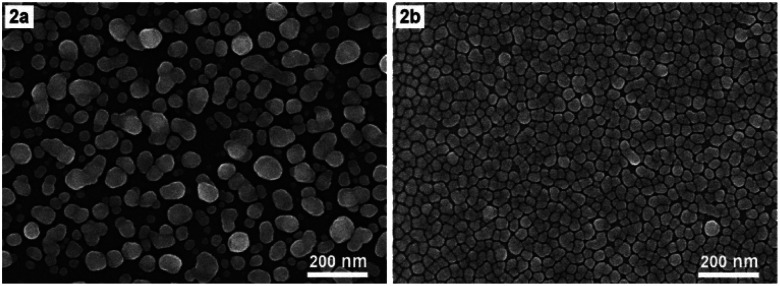
SEM images of AgNPs arrays that have been electrodeposited (with 0.3 mA cm^−2^ current density, at 17 °C, for 4 min) onto Si samples in two different electrolyte solutions (but both contain 0.1 mM AgNO_3_ and 0.14 M HF): aqueous solution (2a), and ethanol solution (2b).

We would like to add that the configuration of a monolayer of uniform and very close AgNPs as shown in Fig. 1b and c is the result achieved not only due to electrodeposition but also thanks to the use of ethanol electrolyte. This fact is illustrated with Fig. 2, which shows the SEM images of AgNPs@Si that have been electrodeposited either in an aqueous electrolyte solution (Fig. 2a) or in an ethanol-based electrolyte solution (Fig. 2b), provided that other conditions are the same (17 °C, 4 min, current density of 0.3 mA cm^−2^, electrolyte solution contains 0.1 mM AgNO_3_ and 0.14 M HF). From Fig. 2a we can see that with the aqueous electrolyte, the formed AgNPs are large and uneven (with sizes spreading in the 20–80 nm region), and they overlap each other, not lying on the same layer. Meanwhile, the picture has changed completely when ethanol electrolyte solution is used (Fig. 2b). Silver nanoparticles become quite uniform in shape and size and they are much smaller (with sizes only of 20–40 nm). The fact that when the ethanol electrolyte was used instead of the aqueous one, the AgNPs became much smaller, suggesting that under new conditions the rate of formation of AgNPs slowed down and became easier to control. Furthermore the silver particles were arranged in a single layer with very small interparticle separation, only a few nanometers. Perhaps it should be recalled that the Raman enhancement factor will only be large enough when the interparticle separation in a SERS substrate is a nanogap.^17^ In addition, the Raman enhancement factor will increase dramatically when this nanogap decreases. For example, for two spherical AgNPs with a diameter of 60 nm, Jiang *et al.* calculated that when the distance between them decreased from 9 nm to 1 nm, the enhancement factor from 1.5 × 10^4^ would increase to 5.5 × 10^9^ (*i.e.* increase up about half a million times).^18^ We believe that the reason for the AgNPs to become more ordered in the arrangement when using ethanol electrolyte instead of aqueous one is that ethanol itself is also a mild reducing agent. The reducing effect of alcohol in general and ethanol in particular has been known for a long time in the synthesis of metal colloids.^19–22^ In the literature in 2014 there was a report of the SERS substrate with AgNPs on copper foil synthesized through a galvanic replacement reaction, in which the ethanol solution of AgNO_3_ has been used instead of aqueous solution.^23^ But unfortunately, in that report the authors did not obtain an array of uniform and orderly silver nanoparticles.

The highly efficient SERS enhancement mechanism of the AgNPs array fabricated in this study can be explained in more detail as follows. As it is well known, the SERS enhancement is determined mainly by the significant increase of the electromagnetic field at the “hot spots” of the SERS substrate.^24^ These hot spots, in turn, are usually the areas around the sharp tips or the narrow gaps between the particles of the substrate. The enhancement will grow up as the number of hot spots as well as their “hotness” increases. The hotness of the hot spots will increase either when the sharpness of the tips increases or when the gap between the particles becomes narrower (especially when the gap reaches a region of a few nanometers). In the case of AgNPs arrays, if it is ensured that the spacing between almost all AgNPs is uniformly reduced to the nanoscale level (rather than the spacing between only a part of particles), the enhancement will be increased greatly. This is realized thanks to a dramatic increase in both the number of hot spots as well as their hotness. This is the goal that we would like to achieve when fabricating the AgNPs array with a few nanometers spacing between nanoparticles for SERS substrates. It is also worth noting that with an orderly arranged AgNPs array one could expect a high reproducibility of the SERS substrates made from it.

**Fig. 3:**
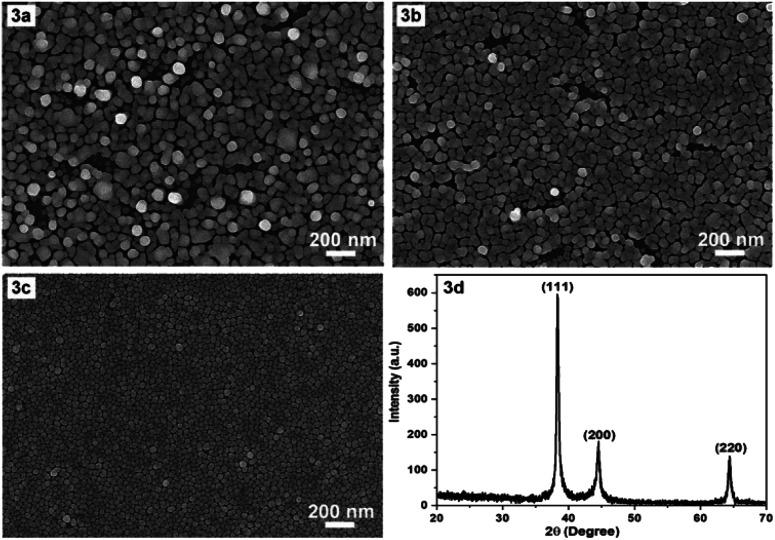
SEM images of AgNPs arrays that have been electrodeposited on Si samples at different temperatures: room temperature (3a), 20 °C (3b), 17 °C (3c) while the other conditions were the same (4 minutes in the ethanol electrolyte solution containing 0.1 mM AgNO_3_ and 0.14 M HF at the 0.3 mA cm^−2^ current density) and a typical XRD pattern of a fabricated AgNPs array (3d).

In the following we would like to present a little more about why a temperature of 17 °C was chosen for the synthesis of AgNPs. It is evident that temperature is a critical factor in the fabrication of AgNPs arrays. The synthesis temperature should be stable and appropriate for obtaining reproducible AgNPs arrays with the desired morphology and configuration. Prior to the chosen 17 °C, room temperature (about 23 °C, non-constant) and constant 20 °C were used for the fabrication of AgNPs arrays with the results illustrated in Fig. 3a and b, respectively. Fig. 3a and b show that the higher the temperature, the larger the size of AgNPs, and the more disordered their arrangement becomes. At 17 °C, AgNPs arranged into a single layer (Fig. 3c), at 20 °C there started to have exceptions, when some AgNPs moved to lie in another layer. At room temperature that was not kept constant, it appeared that the AgNPs lay messily on several different layers. A configuration with cluttered AgNPs is unlikely to guarantee reproducibility of the AgNPs array compared to an ordered arrangement. In addition, the synthesis at 17 °C produced a fairly uniform and very narrow gap, in the nanometer region, between the AgNPs, so this temperature was chosen as the optimal temperature. Next, to ensure that what was formed on the silicon surface was indeed crystalline silver (in the form of nanoparticles), X-ray diffraction (XRD) sampling of this thin film was performed. A typical result is shown in Fig. 3d. The XRD pattern in Fig. 3d shows (111), (200) and (220) peaks of the face-centered cubic silver crystal. The strong and sharp diffraction peaks indicate that silver is well crystallized. No other impurity peaks were detected, indicating the high purity of the as-obtained samples. Thus, the XRD results demonstrate that what was produced after fabrication was definitively silver, in crystalline form.

**Fig. 4:**
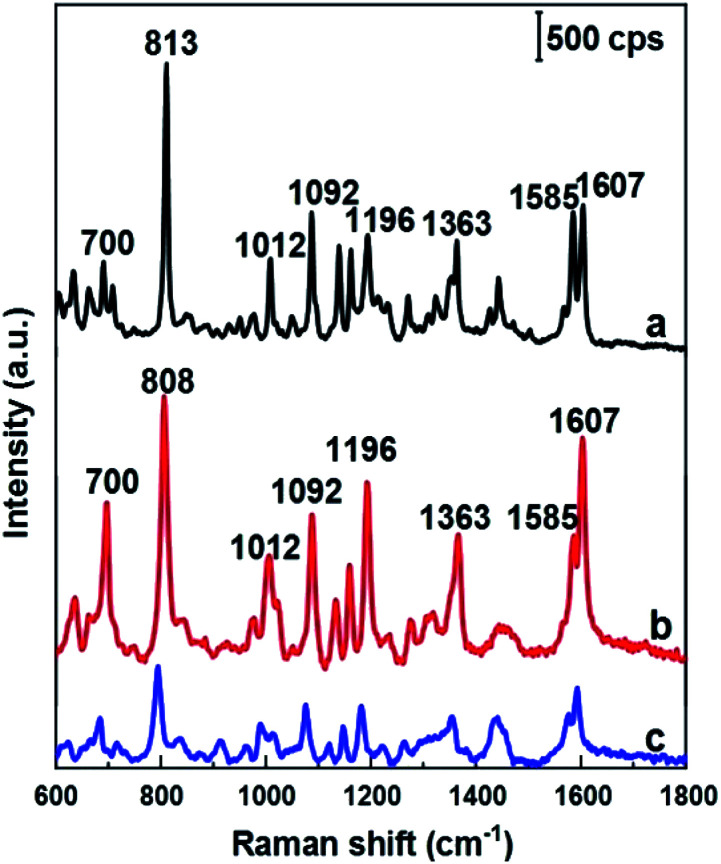
Raman spectrum of DFC powder (a), and SERS spectra of 100 ppm DFC solution adsorbed on SERS substrates, which are AgNPs electrodeposited on silicon in either ethanol electrolyte (b) or aqueous electrolyte (c), provided that other conditions are the same (17 °C, 4 minutes, the electrolyte solution contains 0.1 mM AgNO_3_ and 0.14 M HF, the current density is 0.3 mA cm^−2^).

As mentioned above, DFC is an important fungicide widely used in agriculture, so its molecules have been chosen as the probe molecules to evaluate AgNPs@Si arrays in the role of SERS substrate. For this purpose, AgNPs arrays that have been electrodeposited on Si in the ethanol electrolyte at the current density of 0.3 mA cm^−2^ were used, because they have the best uniformity and the smallest distance between AgNPs. The results of this evaluation are shown in the curve b of Fig. 4, where the SERS spectrum of 100 ppm (2.5 × 10^−4^ M) DFC solution adsorbed onto an AgNPs@Si array is presented. For comparison, AgNPs@Si electrodeposited under the same conditions but in the aqueous electrolyte was also used as the SERS substrate, with the SERS spectrum of 100 ppm DFC adsorbed onto this substrate is shown on the curve c of Fig. 4. It can be seen that the AgNPs@Si substrate fabricated in ethanol electrolyte gives a DFC spectrum with a much higher intensity and a better resolution than the AgNPs@Si substrate fabricated in an aqueous electrolyte. In Fig. 4, the Raman spectrum of DFC powder is also shown (curve a). From the curve a of Fig. 3 we can observe that DFC has characteristic bands at 700, 813, 1012, 1092, 1196, 1363, and 1607 cm^−1^. These characteristic bands are consistent with the results of other authors^13,14^ and the assignment of observed bands to the different vibration modes of DFC is summarized in Table 1. By comparing the curves shown in Fig. 4, we can also see that the SERS spectrum of DFC is in general consistent with its conventional Raman spectrum, with the exception of some relatively small peak shifts and some variation in peaks intensity. For example the peak at 813 cm^−1^ was shifted to the position of 808 cm^−1^. Besides, the intensity of the bands around 700, 1196 and 1607 cm^−1^ has been enhanced remarkably. In general, changes in the SERS spectrum in comparison with its corresponding Raman spectrum can be explained by the interaction of the analyte molecules with the metal nanoparticles of the SERS substrate.^25,26^

**Table 1:** Band assignments of major peaks in the Raman spectrum of difenoconazole^a^^13^

	Assignment
700	*ν*(3-Chloro-4-phenyl-4-chlorophenyl ether)
813	*ν*(3-Chloro-4-phenyl-4-chlorophenyl ether)
1012	*ν*_as_(C–O–C) of chlorophenyl ether, *ν* (3-chloro-4-phenyl)
1092	*ν*(C–Cl) of 4-chlorophenyl, *ν* (4-chlorophenyl)
1138	*ν*(C–N), *ρ*(C–H)
1161	*ν*(C–O) and *ρ*(C–H) of 4-chlorophenyl
1196	*ν*_s_(C–O–C), *ν*(3-chloro-4-phenyl-4-chlorophenyl ether)
1363	*ν*(C=N), *ν*(C–N), *ρ*(C–H), *τ*(CH2)
1585	*ν*(C=C), *ν*(C–C)
1607	*ν*(C=C), *ν*(C–C)

a *ν*—stretching; *ν*_s_—symmetric stretching, *ν*_as_—antisymmetric bending; *ρ*—in-plane bending, *τ*—out-plane bending

**Fig. 5:**
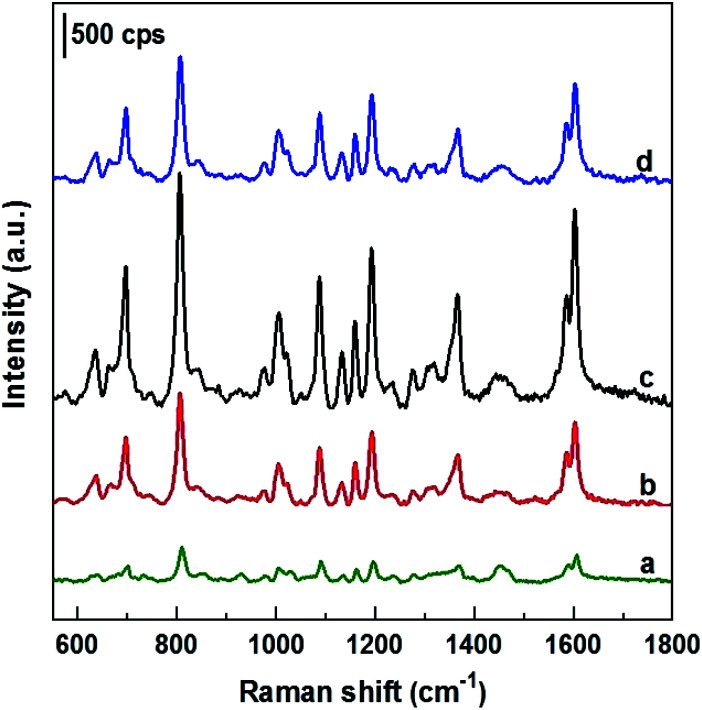
SERS spectra of 100 ppm DFC adsorbed onto SERS substrates which are arrays of AgNPs deposited on silicon in ethanol solution at current densities of 0 mA cm^−2^ (curve a), 0.1 mA cm^−2^ (curve b), 0.3 mA cm^−2^ (curve c), and 0.5 mA cm^−2^ (curve d).

To test SERS performance, AgNPs arrays obtained by deposition in ethanol solutions with different current densities of 0, 0.1, 0.3 and 0.5 mA cm^−2^ (with their SEM images shown in Fig. 1) were used as SERS substrates to detect DFC at the same concentration of 100 ppm. The collected SERS spectra are illustrated in Fig. 5, where curves a, b, c, and d are the spectra of 100 ppm DFC recorded using AgNPs@Si SERS substrates fabricated at current densities of 0, 0.1, 0.3 and 0.5 mA cm^−2^, respectively. It can be seen that the AgNPs@Si substrate fabricated with a current density of 0.3 mA cm^−2^ gives a DFC spectrum with the highest intensity (curve c). Next, AgNPs@Si substrates fabricated at 0.1 mA cm^−2^ and 0.5 mA cm^−2^ gave similar SERS enhancement (curves b and d), while the AgNPs@Si substrate prepared by electroless deposition resulted in the worst enhancement (curve a). Thus, the AgNPs array with the most ordered and nearest AgNPs (obtained at 0.3 mA cm^−2^) also corresponds to the best SERS spectrum of 100 ppm DFC. Therefore, 0.3 mA cm^−2^ was chosen as the optimal current density for AgNPs electrodeposition.

**Fig. 6:**
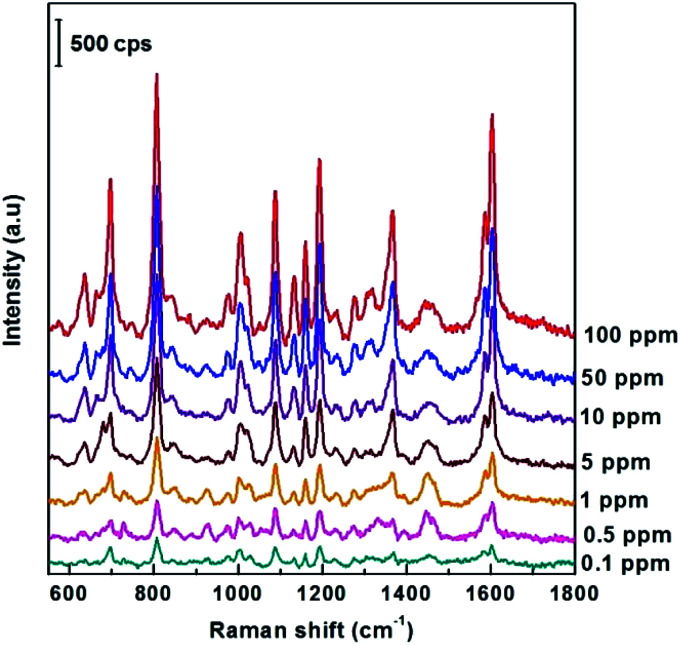
SERS spectra of DFC with concentrations from 0.1 to 100 ppm adsorbed onto AgNPs@Si substrates, which were synthesized by electrodeposition in ethanol electrolyte with the same procedure (17 °C, 4 minutes, the electrolyte contains 0.1 mM AgNO_3_ and 0.14 M HF, the current density is 0.3 mA cm^−2^).

Next, the ability to detect DFC pesticides in the ppm region of the SERS AgNPs@Si substrate synthesized in ethanol electrolyte (at the optimal current density of 0.3 mA cm^−2^) was systematically investigated. This study is illustrated by Fig. 6, which shows the SERS spectra of DFC with concentrations from 0.1 to 100 ppm (2.5 × 10^−7^ M − 2.5 × 10^−4^ M) adsorbed onto the AgNPs@Si substrates mentioned above. From Fig. 6 we can observe that the intensity of the main peaks of the DFC decreases steadily as the concentration of DFC decreases. This fact proves that AgNPs@Si samples have been synthesized with high reproducibility. It should also be noted that the synthesized SERS substrate can detect down to 0.1 ppm of DFC, because at this concentration some of the major peaks of DFC are still clearly visible (see Fig. 6).

**Fig. 7:**
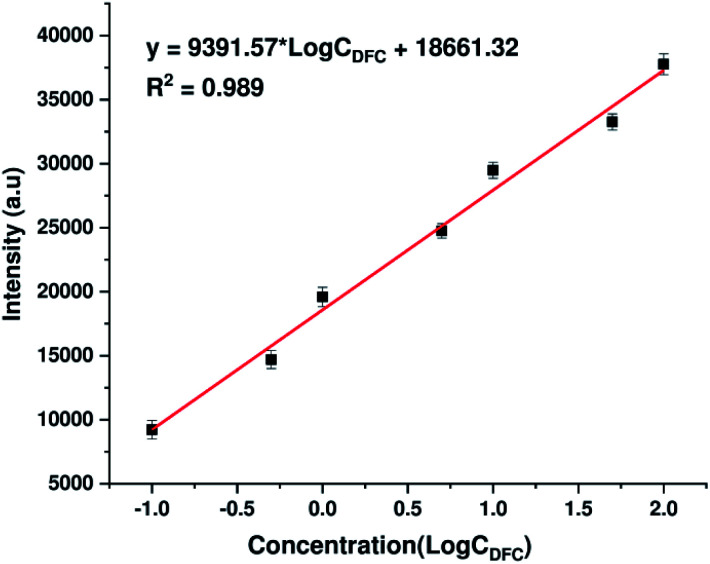
The calibration curve which represents the dependence of the intensity of the Raman band at 808 cm^−1^ of DFC on its concentration in the range of 0.1–100 ppm (expressed in the log scale).

To further assess quantitatively the ability to detect DFC traces of AgNPs@Si substrates synthesized by electrodeposition in ethanol electrolytes, the calibration curve showing the dependence of the intensity of a typical DFC Raman band (specifically the peak at 808 cm^−1^) on its concentration (within the range of 0.1–100 ppm) has been developed and shown in Fig. 7. The peak at 808 cm^−1^ was chosen to represent DFC because it is a single peak with the highest intensity in the SERS spectrum of DFC. As shown in Fig. 7, the dependence of the intensity of the 808 cm^−1^ peak on the DFC concentration in the range of 0.1–100 ppm (on a log scale) is a good linear relationship. The regression equation is *y* = 9391.57 × log *C*_DFC_ + 18 661.32 with the correlation coefficient (*R*^2^) of 0.989, where *y* is the SERS intensity of the peak at 808 cm^−1^ of DFC, and *C*_DFC_ represents DFC concentration. Note that each point in Fig. 7 is the result of averaging the five measurements at different points on the same sample. The error bars represent the standard deviations of five independent measurements. Furthermore, the detection limit of DFC is 0.023 ppm (5.6 × 10^−8^ M), estimated by the signal-to-noise ratio of 3 (S/N = 3). These results indicated the possibility of using AgNPs@Si SERS substrates to quantify trace amount of DFC.

**Fig. 8:**
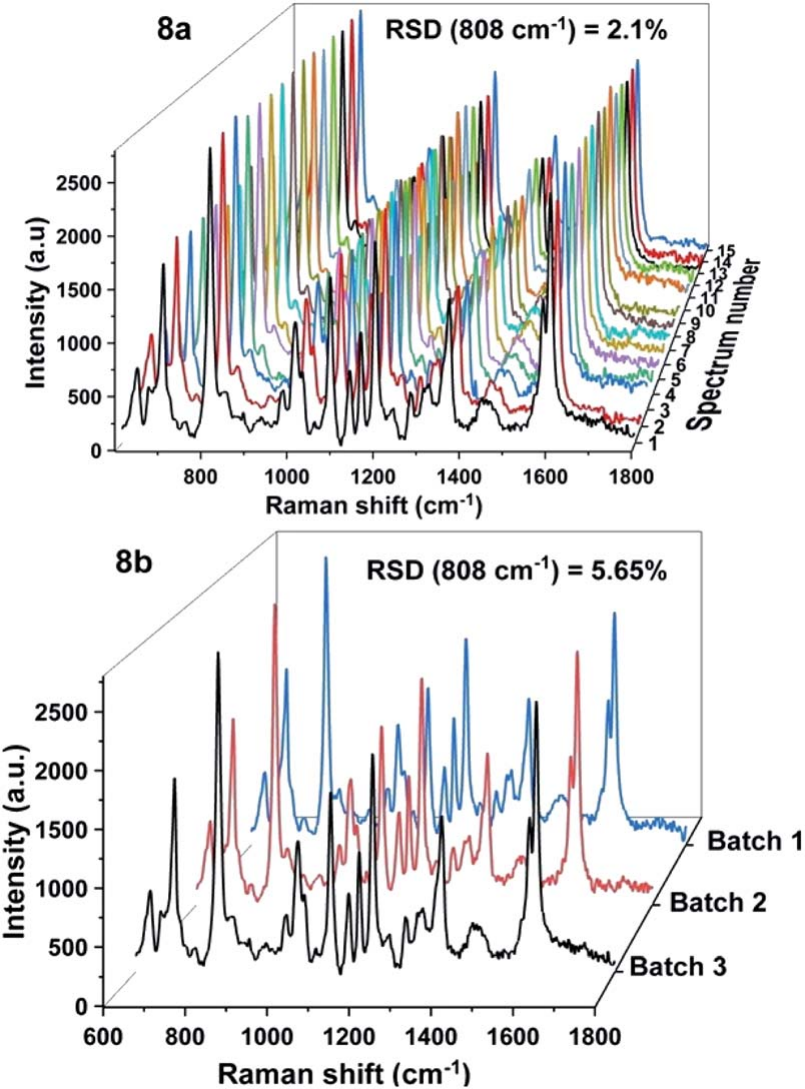
SERS spectra of 100 ppm DFC, which were recorded from 15 random spots on the same AgNPs@Si substrate (8a) and from three AgNPs@Si substrates fabricated in three different batches (8b).

In addition to the sensitivity, uniformity and reproducibility are also essential parameters that determine the reliability of a SERS substrate in applications. To evaluate the prepared SERS substrate in terms of spot-to-spot uniformity, SERS spectra of 100 ppm DFC obtained from 15 different spots on the same AgNPs substrate were recorded and demonstrated in Fig. 8a. Meanwhile, to evaluate the fabricated SERS AgNPs substrates in batch-to-batch reproducibility, SERS spectra of 100 ppm DFC were collected by three AgNPs substrates synthesized in three different batches and the results are shown in Fig. 8b. In general, to evaluate a SERS substrate for uniformity or reproducibility with respect to a particular analyte, one or more bands of the analyte are selected, then the variation in the intensity of the Raman signal of these bands is determined. The variation in intensity of the Raman signal is often expressed in terms of relative standard deviation (RSD). It is generally accepted that the RSD of less than 20% for the variation in the Raman signal intensity is an indicator for a SERS substrate with good uniformity or reproducibility.^27^ In this study the characteristic band of the DFC at 808 cm^−1^ was selected to examine the variation of the Raman signal intensity. Calculations showed that for 15 random spots on the same AgNPs substrate (Fig. 8a) the Raman intensity of the band at 808 cm^−1^ varied with RSD of 2.1%. Thus, the spot-to-spot uniformity of the fabricated AgNPs@Si SERS substrate is very high. Whereas for AgNPs synthesized in three different batches (with 100 ppm DFC spectra obtained when using them as SERS substrates shown in Fig. 8b) we have calculated that the peak intensity at 808 cm^−1^ has fluctuated with RSD of 5.6%. This is also a very positive result.

**Fig. 9:**
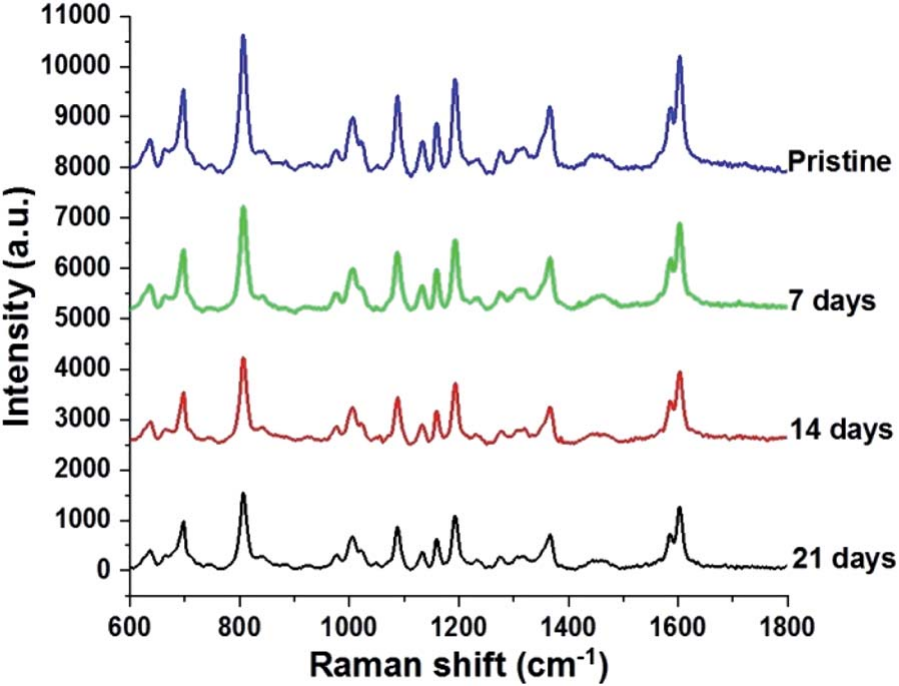
SERS spectra of 100 ppm DFC adsorbed onto different but synthesized with the same procedure AgNPs@Si samples that were stored in air, at room temperature for different time periods, including 0, 7, 14 and 21 days.

Since stability is also an important factor in evaluating a SERS substrate, this feature of the fabricated AgNPs@Si SERS substrate has also been investigated. For this purpose three arrays of AgNPs@Si were prepared on the same day. They are then left in the air at room temperature and used as SERS substrates to record the spectrum of 100 ppm DFC after different time periods, namely 0, 7, 14 and 21 days, respectively. The spectra collected as shown in Fig. 9 demonstrate that the intensity of the main bands of the DFC decreases slightly but steadily with the time of keeping the AgNPs@Si samples in the air. For example, calculations have shown that the intensity of the characteristic band at 808 cm^−1^ of the DFC has decreased with an RSD of 12.23%, 18.65% and 20.43% after 7, 14 and 21 days of AgNPs@Si samples storage in air, respectively. The fact that the Raman signal intensity decreased only about 20% after 21 days of storage in air indicates that the AgNPs@Si SERS substrate synthesized by the method of this study is quite stable with time. In the literature it has been reported that there is a SERS substrate made of silver nanosheets that holds 90% of the intensity of the Raman signal after one month of air storage.^28^ However, in many other reports, the stability of SERS substrates made from AgNPs is much lower (after 20 days of keeping the SERS substrate in the air, the intensity of the Raman signal drops to about 25–30%) and that fact is attributed to the oxidation and sulphurization of silver in air.^29,30^

Although there are many advantages, it should be noted that the SERS substrate constructed according to this study cannot avoid limitations. The first obvious limitation is that the fabrication of the best AgNPs@Si samples requires quite strict conditions. More specifically, the silicon must be of the chosen type, the deposition must be electrochemical, the AgNPs synthesis temperature must be kept constant and the electrolyte solution must be ethanol-based, not aqueous. Meanwhile, the shape, size and arrangement of the AgNPs have yet to achieve the full repetition of the photolithography technique. This will certainly affect the reproducibility of the SERS substrate. Another weakness of the SERS substrate made from AgNPs@Si according to this study is that the silver nanoparticles have an almost spherical or slightly elongated shape, so they do not have sharp tips or protrusions, which are often required to achieve a high SERS enhancement.

## Conclusions

Normally, by immersing a silicon wafer into an aqueous solution containing AgNO_3_ and HF, AgNPs will form and adhere to the surface of the silicon wafer, while part of the silicon surface is etched away with dissolution into solution. Our research has shown that AgNPs with special properties can be obtained if the above electroless deposition of AgNPs is replaced by an electrochemical deposition, with silicon acting as a cathode while a platinum grid plays the role of the anode, in combination with the use of an electrolyte that still contains AgNO_3_ and HF as before, but is an ethanol-based solution (instead of aqueous). More specifically, providing appropriate electrochemical deposition conditions, these AgNPs are very uniform in shape and size, moreover they are very close to one another, with a separation of just a few nanometers, creating a monolayer. Such a monolayer of AgNPs is well suited for use as a reproducible and stable SERS substrate. Indeed, the use of the aforementioned array of AgNPs on silicon as the SERS substrate to detect trace concentrations of difenoconazole (DFC) – a commonly used fungicide, has shown that DFC can be detected in the region of 0.1–100 ppm with a good linear relationship between the intensity of the peak at 808 cm^−1^ (the highest peak in the SERS spectrum of DFC) and the concentration of DFC (expressed on a log scale). Furthermore, the detection limit of DFC was calculated to be 0.023 ppm (5.6 × 10^−8^ M), a figure no worse than that reported by other authors.

## Conflicts of interest

There are no conflicts to declare.

## Supplementary Material
